# The Roles of MicroRNA-122 Overexpression in Inhibiting Proliferation and Invasion and Stimulating Apoptosis of Human Cholangiocarcinoma Cells

**DOI:** 10.1038/srep16566

**Published:** 2015-12-21

**Authors:** Ning Liu, Fan Jiang, Tian-Lin He, Jun-Kuan Zhang, Juan Zhao, Chun Wang, Gui-Xing Jiang, Li-Ping Cao, Peng-Cheng Kang, Xiang-Yu Zhong, Tian-Yu Lin, Yun-Fu Cui

**Affiliations:** 1 Department of General Surgery, Hainan Provincial people’s Hospital, Haikou 570311, P.R. China; 2 Department of Geratology, Hainan Provincial people’s Hospital, Haikou 570311, P.R. China; 3 Department of Pancreatic Surgery, Changhai Hospital, Second Military Medical University, Shanghai 200433, P.R. China; 4 Department of Interventional, the Third Hospital of PLA, P.R. China; 5 Department of Oncology, Xiangyang Central Hospital, Xiangyang 441021, P.R. China; 6 Department of Oncology, Central Hospital of Wuhan, Tongji Medical College, Huazhong University of Science and Technology, Wuhan 430024, P.R. China

## Abstract

Our study investigated whether microRNA-122 (miR-122) played important roles in the proliferation, invasion and apoptosis of human cholangiocarcinoma (CC) cells. QBC939 and RBE cells lines were chosen and divided into five groups: miR-122 mimic group, anti-miR-122 group, negative control (NC) group, mock group and blank group. MiR-122 expression was measured by qRT-PCR. Roles of miR-122 in cell proliferation, apoptosis and invasion were investigated using MTT assay, flow cytometer and Transwell invasion assay, respectively. MiR-122 expression was lower in CC tissues and QBC939 cell than that in normal bile duct tissues, HCCC-9810 and RBE cells. In both QBC939 and RBE cells lines, miR-122 expression was higher in miR-122 mimic group than that in NC group, mock group and blank group; opposite results were found in anti-miR-122 group. Cell proliferation and invasion were remarkably inhibited in miR-122 mimic group after 48 h/72 h transfection, while apoptotic cells numbers were much greater in miR-122 mimic group; the opposite results were obtained from anti-miR-122 group (all *P* < 0.05). MiR-122 expression was significantly weaker in CC tissues, and miR-122 overexpression might play pivotal roles in inhibiting proliferation, stimulating apoptosis and suppressing invasion of CC cells, suggesting a new target for CC diagnosis and treatment.

Cholangiocarcinoma (CC) is the second commonest primary liver malignancy after hepatocellular carcinoma (HCC) and is generally classified into three forms including intrahepatic cholangiocarcinoma (ICC), hilar cholangiocarcinoma and extrahepatic cholangiocarcinoma (ECC)^1^. CC is defined as a rare and fatal tumor, and the main challenge for treating CC is considered to be the accurate diagnosis at the early stage^2^. Incidence rate and mortality rate of ICC have steadily increased over the world in the past few decades while those of ECC decreased^3^. CC widely occurs in different regions with the highest in Southeast Asia and the lowest in Australia, and the rates are 96 per 100,000 in man and 38 per 100,000 in women^4^. The incidence rate is not controlled by improvements in diagnosis because the clinical characteristics of this cancer is not easy to identify^5^. Patients with respectable CC are usually recommended to take surgical resection and can achieve good prognosis, while the prognosis of advanced CC patients remains very poor^6^. Therefore, researchers are trying to develop novel therapeutic strategies to control CC progression^7^,^8^. MicroRNAs (miRNAs) have been found involved in several biological activities in some diseases, and regarded as potential targets for the diagnosis and treatment of CC^9^.

MiRNAs, the non-coding RNA molecules, act as post-transcription regulators of gene expression by binding to the complementary sequences in the 3′ untranslated region^10^. Each miRNA regulates hundreds of protein coding genes involved in cell proliferation, apoptosis and metastasis, and the overexpression of tumor suppressive miRNAs would help to block cell cycle, suppress tumor angiogenesis, and induce apoptosis^11^. MicroRNA-122 (miR-122) is the most abundantly expressed miRNA in human liver cells, and it plays an important role in suppressing cell proliferation and controlling cell viability by targeting antiapoptotic B-cell lymphoma 2 (Bcl-2) family protein Bcl-2 and activating caspase-3 (http://www.scirp.org/journal/PaperInformation.aspx?paperID=22969). The liver homoeostasis is considered to be closely associated with miR-122 expression level; low expression of miR-122 may cause cirrhosis and advanced HCC^12^. MiR-122 also plays a role in various types of cancer by regulating the expressions of the oncogenic proteins such as disintegrin, metalloproteinase domain 10, transcription factor and receptor tyrosine kinase, which are involved in cell adhesion, migration and invasion^13^. The function of miR-122 as tumor suppressor may inhibit the development of CC by binding to target genes related with tumor cell proliferation, apoptosis, invasion and angiogenesis^14^. In this study, we investigated the role of miR-122 in cell proliferation, apoptosis and invasion in CC.

## Materials and Methods

### Patients

Between January 2002 and January 2012, a total of 57 resected paraffin-embedded CC patients (30 males and 27 females; mean age: 61.04 ± 15.20 years; >65, 33 cases; ≤65, 24 cases; range: 28–79 years) were recruited from the Department of General Surgery, Hainan Provincial people’s Hospital, and 57 specimens were collected from surgical excision or biopsy. In addition, 10 normal bile duct tissues diagnosed under the endoscope from liver transplantation were collected to be the healthy control group. Each of the 57 patients was diagnosed by two experienced doctors. The diagnosis of CC was established by laboratory examination and imaging test, such as ultrasound and computed tomography (CT) scan^15^,^16^. The mean tumor diameter was 6.40 ± 4.01 cm (≤5 cm, n = 31; >5 cm, n = 26). Among all the CC patients, 22 cases had lymph nodes metastasis, and 35 cases didn’t. As for distant metastasis, 9 cases were diagnosed with distant metastasis (major metastasis sites were the liver, peritoneum and the greater retina), and 48 cases didn’t have distant metastasis. According to the degrees of differentiation, there were 33 cases in high-moderate differentiation and 24 cases in low differentiation. Tumor node metastasis (TNM) staging was based on the standards of the International Union Against Cancer/American Cancer Society guideline and the seventh edition of the TNM Classification of Malignant Tumors^17^ (stage I–II, n = 38; stage III–IV, n = 19). All the specimens were fixed with 10% neutral buffered formalin, followed by alcohol dehydration, xylene transparency, and wax dipping, and were finally embedded in serial 3–4 mm paraffin blocks and cut into 0.4 mm-thick slices. The clinical data of each patient were complete. Preoperative chemotherapy and biological treatment were not performed before surgery. This study was approved by the Hainan Provincial people’s Hospital, and carried out in accordance with the approved guidelines of the Hospital and the Declaration of Helsinki^18^. Written informed consents were obtained from all the participants and their families.

### Materials

Cholangiocarcinoma RBE, HCCC-9810, and QBC939 cell lines, were purchased from Cell Bank at the Chinese Academy of Sciences; fetal bovine serum (FBS) (Maverick, Beijing, China); streptomycin (Gibco, Grand Island, NY, USA); Trizol reagent (Molecular Science Research Center Inc., Cincinnati, Ohio); ABI 7500 real-time PCR system (Applied Biosystems, Warrington, United Kingdom); Roswell Park Memorial Institute (RPMI) 1640 medium (GIBCO. Uxbridge, UK); Lipofectin^TM^2000 (Invitrogen, Carlsbad, USA); 3-(4,5-dimethylthiazol-2-yl)-2,5- diphenyltetrazolium bromid (MTT) and dimethyl sulphoxide (DMSO) (Sigma Chemical Co. St. Louis, USA); Matrigel (Corning Inc., Lowell, MA, USA); NCode^TM^ miRNA First-Strand complementary DNA synthesis Kit (Invitrogen, Carlsbad, USA); Fast SYBR^®^ Green Master Mix (Roche Diagnostics GmbH, Mannheim, Germany); Transwell chamber (Corning Costar, Tewksbury, MA, USA) and miR-122 mimic and negative control (NC) (Shanghai GenePharma Co., Ltd, Shanghai, China).

### Cell culture and transfection

RBE, HCCC-9810, and QBC939 cells were cultured in RPMI-1640 medium with 10% FBS, 100 U/mL penicillin and 100 μg/mL streptomycin, and were then stored in a 5% CO_2_ humidified incubator at 37 °C. After reaching 80% confluence, the cells were digested with 0.25% pancreatin-ethylene diamine tetraacetic acid diluted in the ratio of 1:5.

### RNA extraction and quantitative real-time polymerase chain reaction (qRT-PCR)

CC specimens were gently crushed under liquid nitrogen and were then collected. Subsequently, cells were all harvested after 48 h of transfection, and the total RNA was extracted with the use of Trizol reagent. RNA purity and concentration were measured by UV spectroscopy. RNA integrity was evaluated using agarose gel electrophoresis. cDNA template was got by reverse transcription (RT) step using NCodeTM miRNA First-Strand complementary DNA synthesis kit, and then Fast SYBR Green Master Mix was used for real-time PCR with ABI 7500 real-time PCR system. Primer sequences for miR-122 were as follows. Forward primer: 5′-TTGAATTCTAACACCTTCGTGGCTACAGAG-′3; reverse primer: 5′-TTAGATCTCATTTATCGAGGGAAGGATTG-′3. U6 was used as internal control and the primer sequences were as follows. Forward primer 5′-CTCGCTTCGGCAGCACA-′3; reverse primer: 5′-AACGCTTCACGAATTTGCGT-′3. Real-time PCR protocol included an initial denaturation step (95 °C for 5 min) and 40 cycles of denaturation (95 °C for 10 s), annealing (60 °C for 20 s) and extension (72 °C for 10 s). The relative expression level of miR-122 was calculated using 2^−ΔΔCt^ method.

### Cells transfection and grouping

The miR-122 expression of different cell lines was measured through qRT-PCR, the result showed that the expression level of miR-122 in QBC93 cells was lower than that in HCCC-9810 and RBE cells, indicating that the QBC93 cell line was suitable for the following experiment. The reason might be that QBC93 cells were derived from liver metastasis foci of extrahepatic bile duct carcinoma, while HCCC-9810 and RBE cells were all from intrahepatic primary foci. Hence the malignant degree of QBC939 cells was comparatively high. One day before the transfection, RBE cells were seeded in 12-well plates at 0.5 × 10^5^ cells/well. The cells were plated to 60% ~ 80% confluence within 24 hours, and 1 μL green fluorescent protein (GFP) (0.5 μg/μl) were then added to the transfected cells. After 72 h incubation, the expression of GFP was observed under the fluorescence microscope with a fluorescence intensity at 350–420 nm. Detailed grouping information were as follows: the miR-122 mimic group was transfected with miR-122 mimics; the anti-miR-122 group was transfected with miR-122 inhibitors; the negative control (NC) group was transfected with miR-122-NC; the Mock group was transfected with reagent by using Lipofectin^TM^ 2000; no transfection was performed on the Blank group. The sequences of transfected miR-122 mimics, miR-122 inhibitors and miR-122-NC (designed and synthesized by Shanghai GenePharma Co., Ltd) were 5′-UGGAGUGUGACAAUGGUGUUUG-′3, 5′-CAAACACCAUUGUCACACU-′3, and 5′-UUCUCCGAACGUGUCACGUTT-′3 respectively.

### Cell proliferation assay

The proliferation of QBC939 and RBE cells were examined at the logarithmic growth phase. The cells were then recovered by digestion with 0.25% trypsin for 20 min, then a volume of RPMI-1640 containing 10% (v/v) FBS was used to terminate the digestion and to suspend the cells with a concentration of 2 × 10^5^ cells/mL. After the incubation in a 24-well plate (500 μL/well), cells were grouped and transfected when these cell lines reached 80% confluency, and three parallel wells were measured in each group. After a 24 h transfection, 1 mL medium was used to culture the suspensions, and then the suspensions were transferred into the 96-well plates after a 1:5 dilution; six parallel wells were measured in each group. After 24 h, 48 h and 72 h of transfection, 10 μl MTT (5 mg/mL) was added to each well, and then the cells were incubated for 4 h in a 5% CO_2_ at 37 °C^19^. Subsequently, the crystallization (purple-red) was observed under the inverted microscope. After the supernatant was discarded, 100 μl DMSO was added to each well, and the mix was shaken for 15 min. The concentrations after 24 h, 48 h and 72 h were measured by absorbance of 570 nm wavelength with a reference wavelength of 630 nm using enzyme-linked immunosorbent assays. All experiments were repeated for at least three times. The cell growth curve was drawn to show cell proliferation rate.

### Cell apoptosis assay

The transfected QBC939 and RBE cells were collected after 48 h (or 72 h) of transfection. Cells were fixed in 70% ethanol for 24 h and centrifuged once. Then DNA-binding dye propidium iodide (DAPI) (10 μg/ml) was added to the cell suspensions for staining, and the mixed solution was incubated at room temperature for 5 min in the dark. Then the samples were observed using the fluorescence microscope. Additionally, the transfected cells after 48 h were washed for three times by cold PBS and fixed in 70% ethanol. After fixation, the cell suspensions were stained in 500 μl precooled 1×binding buffer containing 5 μl annexin V/propidium iodide medium and 2.5 μl propidium iodide. Cell apoptosis was examined on FACScan flow cytometer (Becton-Dickinson, Sunny-vale, CA, USA)^20^.

### Cell invasion assay

At 48 h after the cell transfection, cells were added to 1.5 ml EP tube and centrifuged at 2000 rpm for 5 min. Then the supernatant was discarded and the cells were re-suspended in 200 μl serum-free Dulbecco’s modified Eagle’s medium. The prepared 1 × 10^5^cell was added to the upper chamber coated with Matrigel (8 μm pore size) and polycarbonate membrane. RPMI1640 medium with 10% FBS was placed in the lower chamber^21^. After 24 h at 37 °C, the filters with the mixture of methanol and acetic acid (3:1) were fixed for 30 min, and the cells were stained for 15 min with crystal violet solution. Invasive cells (five high-power fields) were counted using a light microscope.

### Statistical analysis

All the experiments were repeated for three times independently. Enumeration data comparisons were conducted with *I*^2^ test. Numerical data were presented as means ± standard deviation. The comparisons between groups were performed using t test; comparisons among multiple groups were conducted using One-way analysis of variance (ANOVA). Robust tests of homogeneity of variances were conducted before the analysis. Interactions among multiple groups were tested by Fishers-LSD t-tests. SPSS 19.0 software (SPSS, Chicago, IL) was applied for statistical analyses, and *P* < 0.05 was considered to be statistically significant.

## Results

### Expression of miR-122 in CC tissues and cell lines

**Figure 1:**
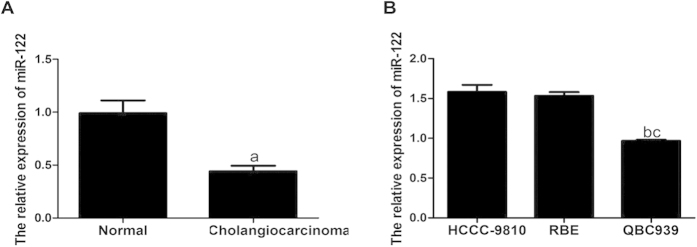
Quantitative real-time polymerase chain reaction (qRT-PCR) detected the relative expression of microRNA-122 (miR-122) in cholangiocarcinoma (CC) tissues and cell lines. (**A**) Comparison of the relative expression of miR-122 between CC tissues and normal bile duct tissues; (**B**) Comparison of the relative expression of miR-122 among three difference cell lines, HCCC-9810, RBE and QBC939. ^a^compared to the normal bile duct tissues, *P* < 0.05; ^b^compared to HCCC-9810 cell line, *P* < 0.05; ^c^compared to RBE cell line, *P* < 0.05.

As shown in Fig. 1A, qRT-PCR results indicated that the expression of miR-122 was significantly lower in CC tissues than that in the normal bile duct tissues, and the relative expression levels (2^−ΔΔCt^) were (0.442 ± 0.051) and (0.990 ± 0.121), respectively (*t* = 14.10, *P* < 0.001). The relative expressions of miR-122 in HCCC-9810, RBE and QBC939 were (1.582 ± 0.088), (1.534 ± 0.047), and (0.968 ± 0.012), respectively. Furthermore, the expression level of miR-122 in QBC939 was significantly lower than those of the first two cell lines (both *P* < 0.05), while there was no significant difference in the expression levels of miR-122 between HCCC-9810 and RBE (*P* > 0.05) (Fig. 1B).

### Association of miR-122 expression with the clinicopathological features of CC patients

The associations between miR-122 expression and the clinicopathological features of the CC patients were presented in Table 1. The results showed that there was no significant association between miR-122 expression and gender, age, tumor size or differentiation (all *P* > 0.05). However, the expression of miR-122 was found significantly associated with lymph node metastasis and distant metastasis (both *P* < 0.05).

### Transfection efficiency of miR-122 in CC QBC939 and RBE cell lines

**Figure 2:**
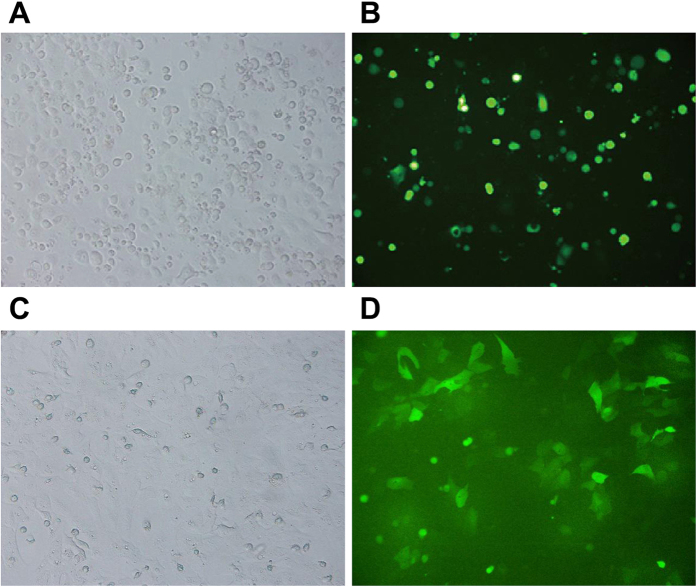
The transfection efficiency of microRNA-122 (miR-122) observation in cholangiocarcinoma under fluorescence microscope. (**A**) QBC939 cells before transfection under fluorescence microscope; (**B**) QBC939 cells after transfection under fluorescence microscope; (**C**) RBE cells before transfection under fluorescence microscope; (**D**) RBE cells after transfection under fluorescence microscope.

The transfection efficiency of GFP was observed by fluorescence microscope for many times for this kind of transient transfection assay, and results indicated that the transfection efficiency of liposome miR-122mimics/inhibitor and Lip2000 in QBC939 and RBE cells were both better than those in the other cells, with the transfection efficiency reached to 70%–80% (Fig. 2).

### MiR-122 expression level after transfection

**Figure 3:**
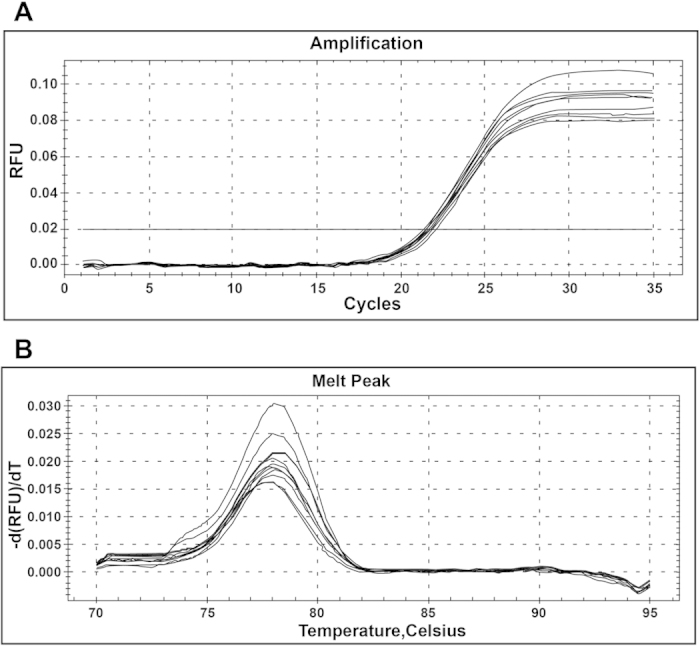
(**A**) The amplification curve of target gene miR-122; (**B**) The dissolution curve of target gene miR-122.

**Figure 4:**
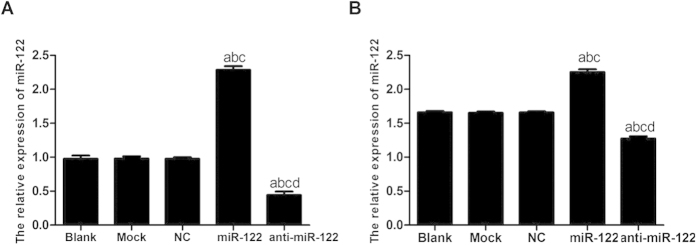
Quantitative real-time polymerase chain reaction (qRT-PCR) testing microRNA-122 (miR-122) expression level after transfection in different groups, (**A**) QBC939 cells; (**B**) RBE cells. NC: negative control; ^a^compared to the blank group, *P* < 0.05; ^b^compared to the mock group, *P* < 0.05; ^c^compared to the NC group, *P* < 0.05; ^d^compared to the miR-122 mimic group, *P* < 0.05.

The amplification curve of target gene miR-122 was in accordance with the smooth curve with four characteristic stages (baseline period, exponential phase, exponential phase, platform stage). As shown in Fig. 3, the dissolution curve had only one peak and no impurity peak, suggesting that the gene was effectively amplified with a single amplification product, which met the experiment requirement. After the transfection with miR-122 mimics, the miR-122 expression levels in QBC939 and RBE cells of the NC group, the Mock group and the Blank group were significantly lower than that of miR-122 mimic group (all *P* < 0.05). However, the expression levels of miR-122 in QBC939 and RBE cells of the NC group, the Mock group and the Blank group were significantly higher than that of anti-miR-122 group (all *P* < 0.05). No significant difference in miR-122 level was found among the NC group, the Mock group and the Bland group (all *P* > 0.05) (Fig. 4 and Table 2).

### The role of miR-122 in inhibiting CC cell proliferation

**Figure 5:**
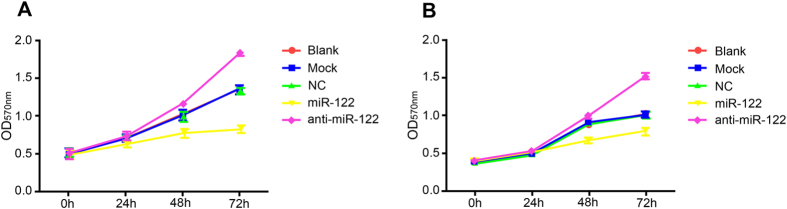
3-(4,5-dimethylthiazol-2-yl)-2,5- diphenyltetrazolium bromid (MTT) assay detected the role of microRNA-122 (miR-122) in inhibiting QBC939 and RBE cell proliferation, (**A**) QBC939 cells; (**B**) RBE cells. NC: negative control; OD, optical density.

At 24 h after co-culture, QBC939 and RBE cell proliferation had no significant difference among the groups (all *P* > 0.05). However, compared to the NC group, Mock group and Blank group, QBC939 and RBE cell proliferations were both significantly inhibited in the miR-122 mimic group at the time points of 48 h and 72 h after the transfection (*P* < 0.05), while the cell proliferation was remarkably promoted in the anti-miR-122 group (Tables 3-4 and Fig. 5).

### The role of miR-122 in stimulating CC cell apoptosis

**Figure 6:**
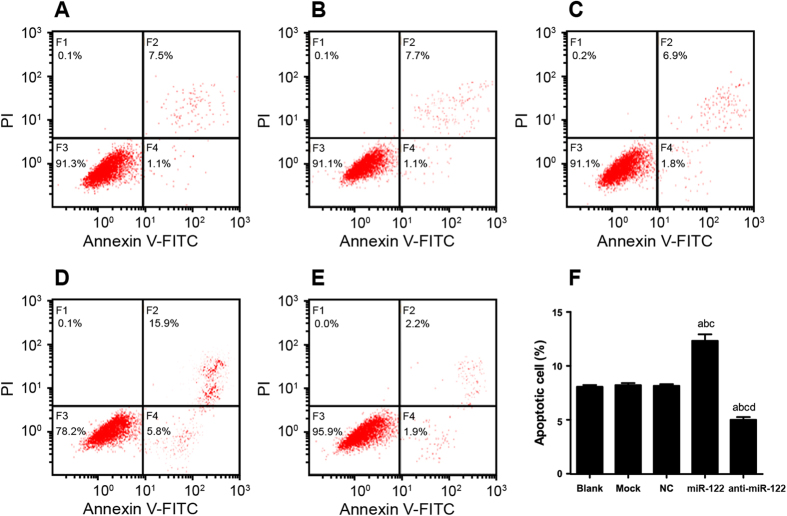
The apoptosis of QBC939 cells stimulated by microRNA-122 (miR-122) detected by flow cytometry in (**A**) Blank group, (**B**) Mock group, (**C**) NC group, (**D**) miR-122 mimic group, (E) anti-miR-122 group; (F) Histogram results of the apoptotic QBC939 cells of different groups. NC: negative control; ^a^compared to the blank group, *P* < 0.05; ^b^compared to the mock group, *P* < 0.05; ^c^compared to the NC group, *P* < 0.05; ^d^compared to the miR-122 mimic group, *P* < 0.05.

**Figure 7:**
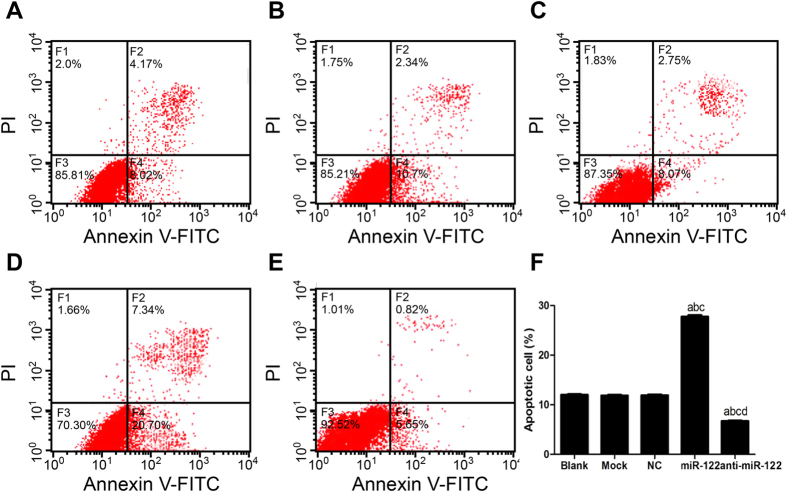
The apoptosis of RBE cells stimulated by microRNA-122 (miR-122) detected by flow cytometry in (**A**) Blank group, (**B**) Mock group, (**C**) NC group, (**D**) miR-122 mimic group, (**E**) anti-miR-122 group; (**F**) Histogram results of the apoptotic RBE cells of different groups. NC: negative control; ^a^compared to the blank group, *P* < 0.05; ^b^compared to the mock group, *P* < 0.05; ^c^compared to the NC group, *P* < 0.05; ^d^compared to the miR-122 mimic group, *P* < 0.05.

As shown in Fig. 6, the rates of QBC939 cell apoptosis in the NC group, the Mock group and the Blank group were (8.76 ± 0.15) %, (8.72 ± 0.18) % and (8.57 ± 0.16) % respectively, with no significant difference between any two (all *P* > 0.05). Similar results were also observed with respect to the rate of RBE cell apoptosis in the NC group (11.91 ± 0.15) %, the Mock group (11.87 ± 0.11) % and the Blank group (12.01 ± 0.13) %, showing no apparent statistically difference (all *P* > 0.05) (Fig. 7). Besides, in the miR-122 mimic group, the rate of QBC939 cell apoptosis was (21.63 ± 0.60) %, and in the anti-miR-122 group, the rate of QBC939 cell apoptosis was (4.11 ± 0.25) %, suggesting that rates of apoptosis in the NC group, the Mock group and the Blank group were significantly different from those in the miR-122 and the anti-miR-122 group (all *P* < 0.05). Similarly, the rate of RBE cell apoptosis in the miR-122 mimics group and the anti-miR-122 group was (27.76 ± 0.31) % and (6.71 ± 0.10) %, respectively, which were significantly different from those in the NC group, the Mock group and the Blank group (all *P* < 0.05).

### The role of miR-122 in suppressing CC cell invasion

**Figure 8:**
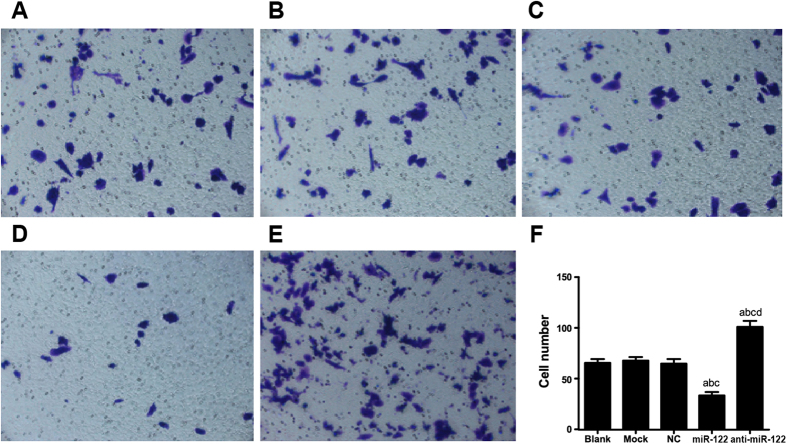
Transwell assay investigated the role of miR-122 in suppressing QBC939 cell invasion in (**A**) Blank group, (**B**) Mock group, (**C**) NC group, (**D**) miR-122 mimic group, (**E**) anti-miR-122 group; (**F**) Histogram results of QBC939 cell invasion detection within different groups. NC: negative control; ^a^compared to the blank group, *P* < 0.05; ^b^compared to the mock group, *P* < 0.05; ^c^compared to the NC group, *P* < 0.05; ^d^compared to the miR-122 mimic group, *P* < 0.05.

**Figure 9:**
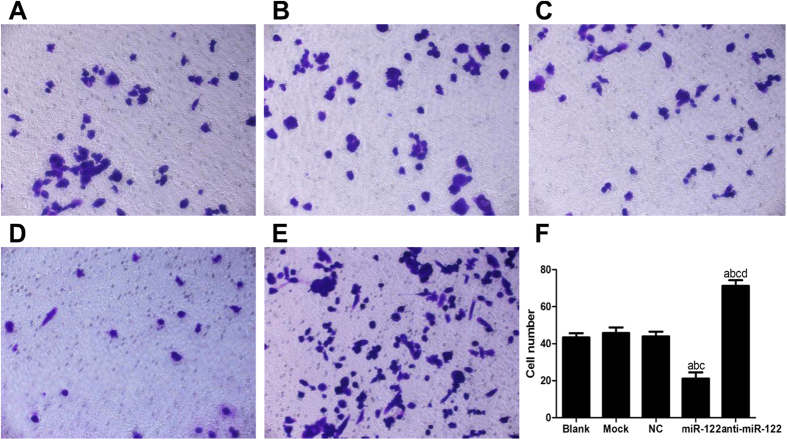
Transwell assay investigated the role of miR-122 in suppressing RBE cell invasion in (**A**) Blank group, (**B**) Mock group, (**C**) NC group, (**D**) miR-122 mimic group, (**E**) anti-miR-122 group; (**F**) Histogram results of RBE cell invasion detection within different groups. NC: negative control; ^a^compared to the blank group, *P* < 0.05; ^b^compared to the mock group, *P* < 0.05; ^c^compared to the NC group, *P* < 0.05; ^d^compared to the miR-122 mimic group, *P* < 0.05.

The cell invasion of QBC939 were measured after 24 h using Transwell invasion assay and Matrigel. The number of QBC939 cells migrating into Matrigel in the miR-122 mimic group (33.47 ± 3.292) was significantly smaller than that in the NC group (64.87 ± 4.42), the Mock group (67.87 ± 3.502) and the Blank group (65.53 ± 3.796) (all *P* < 0.05). However, the number of invasive QBC939 cells into Matrigel in anti-miR-122 group was (101.02 ± 5.92), significantly greater than those in the NC group, the Mock group and the Blank group (all *P* < 0.05). The number of QBC939 cells migrating into Matrigel was not significantly different among the NC group, the Mock group and the Blank group (all *P* > 0.05) (Fig. 8). On the other side, the numbers of RBE cells migrating into Matrigel in the miR-122 mimic group and the anti-miR-122 group were (21.19 ± 3.292) and (71.21 ± 3.091), respectively. In contrast, the number of migrating cells in the miR-122 mimic group was significantly greater than that in the NC group (43.96 ± 2.567), the Mock group (45.76 ± 3.011) and the Blank group (43.42 ± 2.201), while the number in the anti-miR-122 group was significantly smaller than the latter three groups (all *P* < 0.05) (Fig. 9).

## Discussion

MiR-122 serves as functional miRNA involved in regulating lipid metabolism, cell differentiation, hepatic metabolism and hepatitis C virus replication^22^. The design of the experiment constituted a knockout mouse model modeling the loss of miRNA in hepatocytes, and finally liver disruptions including steatosis, inflammation and hepatocyte apoptosis were identified, suggesting that miRNAs played a key role in the liver function and that miR-122 may be associated with hepatocarcinogenesis by influencing hepatocyte survival and tumor progression^23^,^24^. MiR-122 deficiency in CC patients may contribute to the dysregulation of mitochondrial functions related with liver function, so its loss of expression may lead to increased morbidity and mortality and may predict poor prognosis of CC patients^25^. Our study confirmed that the expression of miR-122 was significantly lower in CC tissues than that in normal bile duct tissues, indicating that the decreased expression of miR-122 might be closely related to the development of CC. Therefore, we suggested that regulating the level of miR-122 could be used in controlling the progression of CC, which was consistent with the previous study findings revealing that the modulation of miR-122 level may be one of the targets for the prognosis prediction and the design of effective therapies for CC patients^26^. Furthermore, miR-122 participates in the tumor cell survival, proliferation, differentiation and migration as a tumor suppressor^27^. Previous evidence have shown that compared with adjacent benign liver, miR-122 appears to be down regulated in deficient liver, suggesting the potential of miR-122 as a novel biomarker for liver injury^28^. Further, the magnitude of decrease of the miR-122 level was previously proved to be helpful for the measurement the increase in the grade of hepatocellular injury^29^. Our study also confirmed that the decreased expression of miR-122 was also associated with lymph node metastasis and distant metastasis. Similarly, it was also previously reported that serum miR-122 level was an important indicator in the diagnosis of liver injury and could be used as a biomarker and therapeutic target for diagnosis and treatment of CC-30. In addition, we identified that the expression level of miR-122 in QBC939 was significantly lower than those of HCCC-9810 and RBE cells, while there was no significant difference between HCCC-9810 and RBE. These results supported that QBC939 cells were promising for further *in vitro* experiment; the reason might be that QBC939 cells widely exist in metastatic foci in liver of extrahepatic bile duct carcinoma, while HCCC-9810 and RBE cells mainly exist in the primary foci in the liver, so QBC939 had a higher degree of malignancy^31^.

In our study, we found that the overexpression of miR-122 played pivotal roles in inhibiting proliferation, stimulating apoptosis and suppressing invasion of QBC939 and RBE cells. As the most abundant miRNA in the liver, miR-122 is well known for its biologic function in maintaining liver homeostasis, as well as its role in regulating cell growth, differentiation, apoptosis and metabolism in the carcinogenesis in the liver, which is detrimental to normal liver function^32^,^33^. The over expression of insulin-like growth factor 1 receptor (Igf1R) in the development of tumors stimulates cell growth, survival and proliferation and regulates the initiation of cancer cell metastasis; the level of Igf1R is negatively associated with the level of miR-122 expression, implying that the overexpression of miR-122 can inhibit tumor cell growth and proliferation by suppressing Igf1R expression^34^. MiR-122 also functions as a key modulator of cyclin G1 expression and there is also a negative association between the levels of miR-122 and cyclin G1 (CCNG1)^35^. Decreased level of miR-122 was detected in the CC patients, leading to the increased level of CCNG1, which is associated with accumulation of tumor cells via affecting cell cycle (http://wenku.baidu.com/link? url=qineu6YlskaIZAh01hq9dV1Uw9rC6aU_JUHsgmy_NaJyMuVaIYFt4BErfqFSganqod6GceBfAMuN5rOiL1NZAZ1yikvVs_mgqjoeFy232we). The imbalance between miR-122 and CCNG1 may help to inhibit the tumor cell proliferation of CC through triggering p53 tumor suppressor gene^36^.

A previous study demonstrated that the abnormal expression of miR-122 was responsible for hepatocarcinogenesis; the loss of miR-122 led to the down-regulation of tumor cell apoptosis^37^. MiR-122 expression in tumor cells is suppressed in the early phase of CC, resulting in severe metastasis of tumor cells, and the restoration of its expression may help to control tumor progression of CC patients^38^. As a vital apoptosis regulator, and the mechanism of miR-122 in CC cells involves suppressing Bcl-W mRNA and the protein level, consequently leading to large reduction of cell motility^39^. Bcl-W activity can inhibit cancer cell apoptosis, and the overexpression of miR-122 can inhibit the expression of Bcl-W and CCNG1 to induce cell apoptosis and cell cycle arrest^40^. Thus, down-regulated miR-122 is potential to be an independent predictor of the development and progression of CC characterized by the loss of anti-apoptotic effect^41^. Our study also found that the role of miR-122 in antitumor activity is manifested in suppressing tumor cell invasion. Based on previous study results, miR-122 down-regulation was identified to be associated with hepatic cell invasion, intrahepatic metastasis and reduction of tumor cell sensitivity to drug agent resulting in tumor aggressiveness^42^. As a tumor suppressor, miR-122 can inhibit intrahepatic invasion and migration of CC cells by suppressing angiogenesis through regulating the disintegrin and metalloprotease 17 activity^43^.

In summary, we found that miR-122 expression significantly decreased in CC tissues, and the overexpression of miR-122 played a pivotal role in inhibiting proliferation, stimulating apoptosis and suppressing invasion of CC cells. Finally, our study suggested that miR-122 could be a promising biomarker and target used for the diagnosis and treatment of CC.

## Additional Information

**How to cite this article**: Liu, N. *et al.* The Roles of MicroRNA-122 Overexpression in Inhibiting Proliferation and Invasion and Stimulating Apoptosis of Human Cholangiocarcinoma Cells. *Sci. Rep.* **5**, 16566; doi: 10.1038/srep16566 (2015).

## Figures and Tables

**Table 1:** Association of the relative expression of microRNA-122 (miR-122) and clinicopathological features in cholangiocarcinoma (CC) patients.

Indexes	Cases	Relative expressionof miR-122(2^−ΔΔCt^)	*t*	*P*
Gender				
male	30	0.452 ± 0.051		
female	27	0.430 ± 0.049	1.657	0.103
Age (years)				
≤65	24	0.432 ± 0.044		
>65	33	0.449 ± 0.055	1.250	0.217
Tumor size (cm)				
≤5	31	0.448 ± 0.048		
>5	26	0.433 ± 0.048	1.175	0.245
Histological grade				
well-moderate	33	0.452 ± 0.052		
low	24	0.429 ± 0.048	1.702	0.094
Lymph node metastasis				
with	22	0.392 ± 0.023		
without	35	0.473 ± 0.037	9.195	<0.001
Distant metastasis				
with	9	0.369 ± 0.017		
without	48	0.456 ± 0.043	5.947	<0.001
TNM stage				
I–II	38	0.446 ± 0.050		
III–IV	19	0.434 ± 0.053	0.837	0.406

Note: TNM, tumor node metastasis.

**Table 2:** Quantitative real-time polymerase chain reaction (qRT-PCR) testing microRNA-122 (miR-122) expression level after transfection in different groups.

Group	Relative expression of miR-122 (2^−ΔΔCt^)	
	QBC939	RBE
Blank group	0.977 ± 0.048	1.661 ± 0.020
Mock group	0.978 ± 0.036	1.654 ± 0.017
NC group	0.977 ± 0.022	1.659 ± 0.018
miR-122 mimic group	2.284 ± 0.057^a^^,^^b^^,^^c^	2.254 ± 0.041^a^^,^^b^^,^^c^
anti-miR-122 group	0.442 ± 0.053^a^^,^^b^^,^^c^^,^^d^	1.475 ± 0.033^a^^,^^b^^,^^c^^,^^d^
*F*	693.300	488.600
*P*	<0.001	<0.001

Note: NC: negative control.

^a^compared to the blank group, *P* < 0.05.

^b^compared to the mock group, *P* < 0.05.

^c^compared to the NC group, *P* < 0.05.

^d^compared to the miR-122 group, *P* < 0.05.

**Table 3:** 3-(4,5-dimethylthiazol-2-yl)-2,5- diphenyltetrazolium bromid (MTT) assay detected the role of microRNA-122 (miR-122) in inhibiting QBC939 cell proliferation.

Group	0 h	24 h	48 h	72 h
Blank group	0.494 ± 0.053	0.711 ± 0.047	1.029 ± 0.054	1.360 ± 0.059
Mock group	0.508 ± 0.055	0.705 ± 0.051	1.015 ± 0.075	1.355 ± 0.063
NC group	0.514 ± 0.051	0.690 ± 0.037	0.995 ± 0.067	1.340 ± 0.045
miR-122 mimic group	0.502 ± 0.055	0.637 ± 0.041	0.776 ± 0.059^a^^,^^b^^,^^c^	0.829 ± 0.050^a^^,^^b^^,^^c^
anti-miR-122 group	0.514 ± 0.066	0.752 ± 0.059	1.175 ± 0.026^a^^,^^b^^,^^c^^,^^d^	1.855 ± 0.039^a^^,^^b^^,^^c^^,^^d^
*F*	0.069	2.286	17.860	146.300
*P*	0.990	0.132	<0.001	<0.001

Note: NC: negative control.

^a^compared to the blank group, *P* < 0.05.

^b^compared to the mock group, *P* < 0.05.

^c^compared to the NC group, *P* < 0.05.

^d^compared to the miR-122 group, *P* < 0.05.

**Table 4:** 3-(4,5-dimethylthiazol-2-yl)-2,5- diphenyltetrazolium bromid (MTT) assay detected the role of microRNA-122 (miR-122) in inhibiting RBE cell proliferation.

Group	0h	24 h	48 h	72 h
Blank group	0.371 ± 0.021	0.514 ± 0.029	0.871 ± 0.034	1.017 ± 0.037
Mock group	0.408 ± 0.030	0.505 ± 0.020	0.915 ± 0.031	1.015 ± 0.041
NC group	0.394 ± 0.025	0.497 ± 0.017	0.905 ± 0.037	1.021 ± 0.045
miR-122 mimic group	0.402 ± 0.035	0.521 ± 0.031	0.669 ± 0.041^a^^,^^b^^,^^c^	0.791 ± 0.050^a^^,^^b^^,^^c^
anti-miR-122 group	0.404 ± 0.029	0.534 ± 0.024	1.007 ± 0.039^a^^,^^b^^,^^c^^,^^d^	1.527 ± 0.044^a^^,^^b^^,^^c^^,^^d^
*F*	0.812	1.001	34.970	116.200
*P*	0.546	0.451	<0.001	<0.001

Note: NC: negative control.

^a^compared to the blank group, *P* < 0.05.

^b^compared to the mock group, *P* < 0.05.

^c^compared to the NC group, *P* < 0.05.

^d^compared to the miR-122 group, *P* < 0.05.
